# Knowledge, attitudes and behaviours towards people with HIV and AIDS among private higher education students in Johannesburg, South Africa

**DOI:** 10.4102/sajhivmed.v21i1.991

**Published:** 2020-03-24

**Authors:** Natasha Khamisa, Maboe Mokgobi, Tariro Basera

**Affiliations:** 1 Department of Public Health, School of Engineering, IT, Science and Health, IIE MSA, Johannesburg, South Africa; 2 Department of Psychology, School of Social Science, IIE MSA, Johannesburg, South Africa; 3 Médecins Sans Frontières, Rustenburg, South Africa

**Keywords:** attitudes about contraception, levels of knowledge, risky sexual behaviours, gender differences, young female students

## Abstract

**Background:**

Human immunodeficiency virus and acquired immunodeficiency syndrome (HIV and AIDS) is a global health and social problem, with South Africa having an estimated overall prevalence rate of 13.5%. Compared to young male participants, young female participants have been reported to have less knowledge about HIV and AIDS, including prevention strategies, and this is associated with risky sexual behaviours and negative attitudes towards condom use.

**Objectives:**

The study investigated gender differences in knowledge, attitudes and behaviours towards HIV and AIDS among 542 private higher education students in Johannesburg, South Africa.

**Method:**

Participants completed an online structured questionnaire measuring knowledge, attitudes and behaviours as well as demographics (including age, gender and relationship status).

**Results:**

The results indicate that overall there were no significant differences between male and female students in terms of HIV and AIDS knowledge. However, female students had significantly less knowledge with regard to unprotected anal sex as a risk factor for HIV and AIDS. In addition, young female students reported condom use at last sex less frequently than male students. Nonetheless, both genders reported a positive attitude towards condom use and towards people living with HIV and AIDS.

**Conclusion:**

It is recommended that the relevant authorities at the state and the higher education level seriously consider implementing specific strategies for preventing HIV and AIDS through improved knowledge, attitudes and behaviours among young females.

## Introduction

Since the beginning of the epidemic, human immunodeficiency virus and acquired immunodeficiency syndrome (HIV and AIDS) has affected more than 70 million people globally, accounting for 35 million deaths.^1^ As of 2018, over 30% of the global HIV and AIDS prevalence has been among the youth aged 15–25 years, with 5 million young people currently living with HIV and AIDS.^1^ The youth between the ages of 15 and 24 years account for 45% of new infections.^2^ The burden of HIV and AIDS is concentrated in sub-Saharan Africa, where 71% of people living with HIV reside and where 65% of new infections reported in 2017 occurred.^3^ The female population is disproportionately affected by HIV, with three in four new infections reported among girls aged 15–19 years, while the young female population aged 15–24 years is twice as likely to be living with HIV and AIDS than their male counterparts.^4^

South Africa is known to have one of the highest rates of HIV and AIDS globally and on the African continent. It is estimated that 7.97 million South Africans (13.5%) are living with HIV and AIDS, and over a fifth of these are females of reproductive age (15–49 years). Gauteng Province, the most populous province in the country (25.8% of the population), is home to over 2 million young people, the majority of whom are females.^5^ The high infection rate among the young female population is attributed to a lack of knowledge as well as poor attitudes towards the use of condoms and risky sexual behaviour.^6^ Knowledge regarding HIV and AIDS infection is necessary to correct negative attitudes towards condom use and to encourage healthy sexual behaviour among the youth by improving their ability to practice safe sex. This is likely to improve the uptake of HIV prevention strategies to address the increase in the prevalence of HIV and AIDS in vulnerable populations.^6,7^ Young female participants are twice as likely to be infected with HIV and AIDS, compared to young male participants, with the most common means of transmission being unprotected sex, and the key barriers to prevention being lack of knowledge, negative attitudes and risky sexual behaviour.^3,6,8^

Knowledge facilitates familiarity with and awareness of HIV and AIDS, which influences attitudes (resulting in support and motivation for prevention) as well as behaviour (safer sex practices), thereby reducing the risk of infection.^9^ A high prevalence of HIV and AIDS is associated with lower levels of knowledge about the modes of transmission and condom use, negative attitudes towards condom use and risky sexual behaviours such as unsafe sex and multiple sex partners.^10,11^ The young female population has been shown to possess significantly lower levels of knowledge as well as misconceptions and erroneous beliefs about HIV and AIDS, compared to the male population.^6,12^ This is often associated with negative attitudes to prevention strategies, which have been shown to affect behaviours such as condom use.^13^ Studies have also confirmed that HIV and AIDS testing attitudes and the intention to use condoms are influenced by knowledge.^14,15^

Charles et al.^16^ reveal that although the young female population is more concerned about HIV and AIDS infection, they agree less than the young male population on condom use as a safe sex strategy. The attitudinal gender differences of the young female population with regard to transactional sex are thought to result in risky behaviours such as unprotected sex with older men.^17^

Although it is known that the young female population is more susceptible to HIV and AIDS infection, there is a paucity of research on gender differences in knowledge, attitudes and behaviours among young people in higher education settings, thereby inhibiting an in-depth understanding of factors contributing to HIV and AIDS infection among this population in South Africa. Such a gap in the literature negatively influences policy and practice aimed at reducing HIV and AIDS rates among vulnerable youth within this context. This study was aimed at identifying gender differences in knowledge, attitudes and behaviours among the youth at a private higher education institution in Johannesburg, South Africa. It is envisaged that this will allow for the development of specific strategies for preventing HIV and AIDS infection among the youth at private higher education institutions in South Africa. The research question seeks to determine differences between the male and female populations on knowledge, attitudes and behaviours towards HIV and AIDS. It is hypothesised that knowledge, attitudes and behaviours will differ between the male and the female populations.

## Methods

### Study design

This cross-sectional survey was conducted at a private higher education institution in Johannesburg, South Africa. Participants were invited via an online learning platform where they completed and submitted a structured questionnaire assessing sexual risk and sexual prevention behaviours.

### Setting

Johannesburg is the capital of Gauteng province and is considered the largest and the wealthiest city in South Africa. With a population estimated at 5.6 million, it is the most populous city in the country, with 66% growth rate in population expected over the next 30 years. Racial profiles indicate that 76.4% of the population is black African, 5.6% is mixed race, 12.3% is white or of European descent and 4.9% is of Indian or Asian descent. Approximately, 7% of the population is illiterate, 3.4% have only a primary education, 41% have completed secondary education and 6% have a tertiary qualification.^18^

### Study population and sampling

Random sampling was used to recruit 845 students enrolled at a private higher education institution in Johannesburg, South Africa. Random numbers were generated using a computer program to select participants from the sampling frame – enrolment records. A global email was sent to all potential participants inviting them to participate in the study. Of those invited to participate in the study, 542 responded – a response rate of 64%. Participants completed an online questionnaire via their online learning platform, which contained study information and instructions for accessing and completing the questionnaire.

### Data collection and analysis

An online structured questionnaire measuring knowledge, attitudes and behaviours as well as demographics (including age, gender and relationship status) was completed by the participants. The questionnaire was developed as part of a larger study using existing literature and consisted of 93 questions of which 51 questions were on knowledge about HIV and AIDS transmission and prevention, attitudes towards HIV and AIDS, including treatment and prevention methods (six questions), and sexual behaviours (36 questions).

Data were cleaned and checked for errors before coding and analysing using STATA 14.0 (StataCorp, College Station, TX, USA). To evaluate knowledge and behaviours, respondents were required to provide mostly ‘yes’ or ‘no’ responses. For the knowledge score, a score of 1 was assigned for a correct answer and 0 for a wrong answer. For the attitude questions, a rank was assigned using the Relative Importance Index to obtain an overall rank for each attitude item. For knowledge, attitudes and practice questions (knowledge and awareness of HIV & AIDS, prevention and control of HIV, students’ attitudes towards condom use and people living with HIV, risky sexual behaviours), the frequency of responses in each category was determined. A chi-square test was used to evaluate the variation in knowledge, attitude and behaviour between male and female students. For all tests, *p* < 0.05 was considered statistically significant.

### Ethical considerations

Ethical approval to conduct the study was obtained from Monash University Human Research Ethics Committee (MUHREC) (approval number CF15/1095 – 2015000518). No identifying information was obtained from students when they completed the questionnaire. A unique identifier was generated when the questionnaire was submitted. All the data were de-identified for analysis. Data were stored in password-protected files and will be retained for up to 5 years after the study.

## Results

### Socio-demographic characteristics of the students

Data were collected from 542 students, 374 (69.0%) of whom were female students. The participants had a median age of 19 years (interquartile range [IQR] = 16–30 years), and their average knowledge score of HIV and AIDS and sexually transmitted infections (STIs) was 0.78 (standard deviation [SD] = 0.17); 397 (73.2%) students were black Africans, 67 (12.4%) students were whites, 44 (8.1%) students were of Indian/Asian descent and 29 (5.4%) students were of mixed race; 427 (80.6%) students were single and 88 (16.6%) students were in a stable relationship. Most of the students were in the Higher Certificate, Higher Education Studies’ stream (*n* = 357, 71.1%), and 145 (28.9%) students were undergraduates (Table 1).

**TABLE 1:** Socio-demographic characteristics of students.

Variable	*n*	%
**Age (*N* = 542)**		
15–19	375	69.2
20–32	167	30.8
**Sex (*N* = 542)**		
Male	168	31.0
Female	374	69.0
**Population group (*N* = 542)**		
Black African	397	73.2
White	67	12.4
Mixed race	29	5.4
Indian or Asian	44	8.1
Other	5	0.9
**Religion (*N* = 529)†**		
African traditional	16	3.0
Christian	425	80.3
Muslim	25	4.7
Hindu	18	3.4
No religion	40	7.6
Other	5	1.0
**Citizenship (*N* = 542)**		
South African	378	69.7
Non-South African	164	30.3
**Relationship status (*N* = 530)†**		
Single	427	80.6
Going steady	88	16.6
In a relationship or cohabiting	3	0.6
Divorced or widowed	3	0.6
Married	9	1.7
**Living arrangements (*N* = 537)†**		
In a relationship but not living together	80	14.9
Living with boyfriend or girlfriend or partner	13	2.4
Living alone	106	19.7
Living with friends or peers or fellow students or other people	139	25.9
Living with family or relatives	199	37.1
**Locality type (*N* = 540)†**		
Campus accommodation	143	26.5
Off-campus accommodation	397	73.5
**Level of study (*N* = 502)†**		
Higher Certificate in Higher Education Studies	357	71.1
Undergraduate	145	28.9
**Financial situation of household (*n* = 542)**		
Not enough money for basic necessities like food and clothes	10	1.9
Have money for food and clothes, but short of many other necessities	59	10.8
Have most of the important things, but few luxury goods	270	49.8
Have some money for extra things such as going on a holiday and buying luxury goods	203	37.4

† , Missing values.

There were high levels of awareness and knowledge about biomedical methods of HIV prevention amongst the sample group. More female (77.1%) than male students (64.1%, *p* = 0.003) said that they had heard about medication that HIV-positive pregnant women could take to reduce the risk of infecting the baby with HIV, and more female (59.9%) than male students (47.6%, *p* = 0.015) had heard about medication that could help to reduce the risk of HIV infection if a woman had been raped. A higher proportion of male (95.8%) versus female students (85.7%, *p* = 0.001) said that they were able to obtain a condom. There was no significant difference between the proportion of male (97.0%) and female students (94.1%) about where to get condoms (Table 2).

**TABLE 2:** Student’s knowledge of and access to HIV and AIDS prevention and control.

Statement	Female				Male				*p*
	Yes		No		Yes		No		
	*n*	%	*n*	%	*n*	%	*n*	%	
Do you know of a place where a person can get condoms?	350	94.1	22	5.9	163	97.0	5	3.0	0.147
If you wanted to, could you yourself get a condom?	301	85.7	50	14.3	158	95.8	7	4.2	0.001***
Have you heard of drug treatments that HIV-positive pregnant women can take to reduce the risk of infecting the baby?	287	77.1	85	22.9	109	64.9	59	35.1	0.003***
Have you heard of drug treatments that can help reduce the risk of HIV infection if a woman has been raped?	219	58.9	153	41.1	80	47.6	88	52.4	0.015***

HIV, human immunodeficiency virus; AIDS, acquired immunodeficiency syndrome.

*** , significant at *p* < 0.05.

Knowledge about HIV transmission was high, with 98.1% of female students and 96.4% of male students knowing that the virus could be passed on through unprotected sex. A high proportion of male students (92.9%) and female students (90.9%) knew that a person can have HIV and pass it on to others without showing symptoms. Also, most female students (77.4%) and male students (76.8%) knew that STIs put people at greater risk of HIV infection. However, only 33.6% of female students and 39.3% of male students admitted that they knew that anal sex increased the risk of HIV infection. A low proportion of female students (16.9%) and male students (21.4%) knew that people can reduce their chance of getting HIV by using a condom every time they have sex. More female (96.2%) than male students (91.7%) were aware that AIDS could not be cured. Most of the female students (*n* = 307, 82.5%) and male students (*n* = 119, 70.8%) indicated that partners *could not* have sexual intercourse if both partners were HIV-positive (*p* = 0.002) (Table 3).

**TABLE 3a:** Frequency and percentage of students’ knowledge and awareness of HIV and AIDS.

Awareness of AIDS	Female				Male				*p*
	Yes		No		Yes		No		
	*n*	%	*n*	%	*n*	%	*n*	%	
Have you ever heard of AIDS?	350	94.1	22	5.9	157	93.4	11	6.6	0.776

AIDS, acquired immunodeficiency syndrome.

**TABLE 3b:** Frequency and percentage of students’ knowledge and awareness of HIV and AIDS.

Knowledge about HIV and STI transmission	Female				Male				*p*
	True		False		True		False		
	*n*	%	*n*	%	*n*	%	*n*	%	
HIV can spread to males or females through unprotected sex	365	98.1	7	1.9	162	96.4	6	3.6	0.236
If a woman uses birth control pills or injection, it lowers her risk of getting infected with HIV	47	12.6	325	87.4	30	17.9	138	82.1	0.108
STIs put people at greater risk for HIV infection or infection with new forms of the virus	288	77.4	84	22.6	129	76.8	39	23.2	0.871
A person can have the AIDS virus and pass it on to others even if the person does not look sick	338	90.9	34	9.1	156	92.9	12	7.1	0.442
If both partners are HIV-positive, it is okay to have unprotected sex	65	17.5	307	82.5	49	29.2	119	70.8	0.002***
Having anal sex increases your chances of getting infected with HIV	125	33.6	247	66.4	66	39.3	102	60.7	0.201
The HIV virus can be passed from a pregnant mother if she is infected with HIV to her unborn child	312	83.9	60	16.1	139	82.7	29	17.3	0.743
People can protect themselves from getting infected with the HIV virus by not having sexual intercourse	234	62.9	138	37.1	113	67.3	55	32.7	0.328
AIDS can be cured	14	3.8	358	96.2	14	8.3	154	91.7	0.027***
Can people reduce their chances of getting the AIDS virus by using a condom every time they have sex?	63	16.9	309	83.1	36	21.4	132	78.6	0.212

HIV, human immunodeficiency virus; AIDS, acquired immunodeficiency syndrome; STI, sexually transmitted infection.

*** , significant at *p* < 0.05.

As illustrated in Table 4, a substantial number of students expressed positive attitudes towards condom use. Across age and gender groups, a significant majority of students disagreed with the statement that a woman loses a man’s respect if she asks him to use a condom (96.8% of female participants and 91.7% of male participants); that they only use condoms if their sexual partner wants to use them (95.7% female participants and 86.3% of male participants); and that condoms should only be used if having sex with a person who is not the main sexual partner (94.6% of female participants and 83.9% of male participants). About a third of the students and 41.7% of the male students said that condoms felt unnatural, and nearly a quarter of the students aged 20–32 years and 36.3% of the male students said that condoms alter climax or orgasm.

**TABLE 4a:** Student’s attitudes related to condom use.

Variable	Total	If I ask my sexual partner to use a condom, he would think I do not trust him				If I ask my sexual partner to use a condom, he might get angry				If I ask my partner to use a condom, he might get turned off				Condoms feel unnatural				Condoms change the climax or orgasm			
		Agree		Disagree		Agree		Disagree		Agree		Disagree		Agree		Disagree		Agree		Disagree	
		*n*	%	*n*	%	*n*	%	*n*	%	*n*	%	*n*	%	*n*	%	*n*	%	*n*	%	*n*	%
**Age (years)**																					
15–19	375	50	133.3	325	86.7	30	8.0	345	92.0	39	10.4	336	89.6	73	19.5	302	80.5	64	17.1	311	82.9
20–32	165	27	16.4	138	83.6	16	9.7	149	90.3	18	10.9	147	89.1	54	32.7	111	67.3	41	24.9	124	75.1
**Sex**																					
Female	372	50	13.4	322	86.6	32	8.6	340	91.4	36	9.7	336	90.3	57	15.3	315	84.7	44	11.8	328	88.2
Male	168	27	16.1	141	83.9	14	8.3	154	91.7	21	12.5	147	87.5	70	41.7	98	58.3	61	36.3	107	63.7

**TABLE 4b:** Student’s attitudes related to condom use.

Variable	Total	A woman loses a man’s respect if she asks him to use a condom				I use condoms only if my sexual partner wants me to use it				Condoms should only be used if having sex with a person who is not the main sexual partner			
		Agree		Disagree		Agree		Disagree		Agree		Disagree	
		*n*	%	*n*	%	*n*	%	*n*	%	*n*	%	*n*	%
**Age (years)**													
15–19	375	18	4.8	357	95.2	24	6.4	351	93.6	25	6.7	350	93.3
20–32	165	8	4.9	157	95.1	15	9.1	150	90.9	22	13.3	143	86.7
**Sex**													
Female	372	12	3.2	360	96.8	16	4.3	356	95.7	20	5.4	352	94.6
Male	168	14	8.3	154	91.7	23	13.7	145	86.3	27	16.1	141	83.9

Most participants had positive attitudes towards HIV-positive people, but 38 (7%) participants were unwilling to be associated with or share living space with people living with HIV. Based on the relative importance index score, the most important attitudes towards people living with HIV, ranked in order of relative importance, were: (1) About 83.4% of students indicated that even if a family member had HIV, their relationship with them would remain good; (2) 20% of students said that sharing a house with HIV-positive people would be very difficult for them; and (3) 7.6% of students felt that people who get infected with HIV are promiscuous. Forty-one (7.6%) students also said they do not want to be associated with HIV-positive people, and 6.4% of students felt that HIV-negative people should not be allowed to socialise with HIV-positive people. Students had positive attitudes towards treatment for HIV and AIDS. Around two-thirds (63.1%) of the students agreed that HIV treatment would keep an HIV-positive person alive (ranked the most important attitude). Two hundred and seventy-four (50.8%) students agreed that HIV medication really works (with a relative importance score of 2). Most of the respondents (69% students) rejected the notion that antiretroviral (ARV) medication is poisonous (Table 5).

**TABLE 5:** Attitudes of students towards people who are HIV-positive or have AIDS and HIV and/or AIDS treatment.

Statements	Strongly agree		Agree		Neither agree or disagree		Disagree		Strongly disagree		Relative importance index	Rank
	*n*	%	*n*	%	*n*	%	*n*	%	*n*	%		
**Attitudes of students towards people who are HIV-positive or have AIDS**												
I do not want to be associated with HIV-positive people	23	4.3	18	3.3	85	15.7	164	30.4	250	46.3	0.38	4
Even if a family member had HIV, my relationship with them would remain good	298	55.2	152	28.2	48	8.9	15	2.8	27	5.0	0.85	1
Staying in the same house or hostel with HIV-positive people would be extremely difficult for me	38	7.0	70	13.0	140	25.6	117	21.7	175	32.4	0.48	2
People who get infected with HIV are promiscuous	16	3.0	25	4.6	210	38.9	115	21.3	174	32.2	0.45	3
HIV-negative people should not be allowed to socialise with HIV-positive people	22	4.1	13	2.4	52	9.6	67	12.4	386	71.5	0.27	5
**Attitudes of students towards HIV treatment**												
I do not trust that HIV treatment works	23	4.3	26	4.8	165	30.6	173	32.0	153	28.3	0.45	3
HIV treatment will keep an HIV-positive person alive	119	22.0	222	41.1	137	25.4	39	7.2	23	4.3	0.74	1
You can share HIV treatment with your partner	14	2.6	27	5.0	144	26.7	105	19.4	250	46.3	0.40	4
ARVs are poisonous	16	3.0	23	4.3	127	23.5	93	17.2	281	52.0	0.38	5
HIV medicine really works	95	17.6	179	33.2	227	42.0	16	3.0	23	4.3	0.71	2

HIV and AIDS, human immunodeficiency virus and acquired immunodeficiency syndrome; ARV, antiretroviral.

Condom use at last sex was higher when with a regular partner: female students (*n* = 147) and male students (*n* = 93). The difference is statistically significant (*p* < 0.001). Fewer female students (*n* = 53) and male students (*n* = 57) reported using condoms consistently with a non-regular partner (*p* = 0.049). More female students (*n* = 83) reported consistent condom usage (every time) with regular sex partners than male students (*n* = 54); however, the difference was not significant (*p* = 0.240) (Table 6).

**TABLE 6:** Condom use among students.

Variable	Female		Male		*p*
	*n*	%	*n*	%	
**Condom use at last sex with a partner**					0.001***
Yes	147	39.3	93	55.4	
No	227	60.7	75	44.6	
**Consistent condom use with partners**					**0.240**
Every time	83	38.8	54	42.5	
Almost every time	36	16.8	30	23.6	
Sometimes	31	14.5	18	14.2	
Never	31	14.5	11	8.7	
Do not know	33	15.4	14	11.0	
**Condom use at last sex with a non-regular partner**					0.002***
Yes	59	42.7	71	62.8	
No	79	57.3	42	37.2	
**Consistent condom use with a non-regular partner**					0.049***
Every time	53	40.2	57	53.3	
Almost every time	11	8.3	13	12.2	
Sometimes	10	7.6	5	4.7	
Never	17	12.9	15	14.0	
Do not know	41	31.1	17	15.9	
**Condom use at last sex with a commercial sex worker**					0.001***
Yes	57	30.7	55	49.6	
No	129	69.3	56	50.4	
**Consistent condom use with a commercial sex worker**					**0.138**
Every time	49	27.8	41	38.0	
Almost every time	11	6.3	11	10.2	
Sometimes	9	5.1	7	6.5	
Never	38	21.6	15	13.9	
Do not know	69	39.2	34	31.5	

*** , significant at *p* < 0.05.

## Discussion

South Africa is battling an HIV and AIDS pandemic, which remains one of the primary social and health concerns in the country. Notwithstanding the outstanding effort by the Department of Health in implementing HIV and AIDS prevention strategies, South Africa is still the country worst affected by the HIV and AIDS pandemic, with the youth between the ages of 15 and 24 years being the hardest hit.^5^ Young female students are reported to have higher infection rates owing to a lack of knowledge and poor attitudes towards condom use and risky sexual behaviours,^6^ making them twice as likely as male students to be infected with HIV.^3,6,8^ This study was aimed at determining gender differences in knowledge, attitudes and behaviour in relation to HIV and AIDS among students at a private higher education institution in Johannesburg, South Africa.

Findings in this study indicate that there is no significant difference between male and female students in terms of their general knowledge of HIV and AIDS. However, it is noteworthy that female students had significantly less knowledge of unprotected anal sex as a risk factor for HIV and AIDS. In addition, a smaller proportion of female students reported condom use at last sex, compared to their male counterparts. This could be attributed to the female population having limited control over male condom usage.^19^ The complex power imbalance in this scenario, with most females not having the power to negotiate condom use with their partners, is the likely underlying cause.^20,21^

Moreover, 8.3% of male students and 3.8% of female students believed that AIDS can be cured. Although these percentages are comparatively low, they are equally as disconcerting as the results in Haroun et al.,^22^ who found that just over 20% of a sample of university students in the United Arab Emirates did not know whether HIV and AIDS could be cured or not. This poor knowledge of basic messages relating to HIV and AIDS is a likely reason as to why the youth engage in risky sexual behaviour and why the prevalence of HIV infection among them is high.^23^

Regarding risky sexual behaviour, this study revealed notable differences between male and female students, with the latter (57.3%) and the former (37.2%) reporting not having used a condom at last sex with a non-regular partner. As the chi-square test revealed no significant variation in attitudes between male and female students, we opted to investigate the entire sample’s attitudes rather than to compare male and female students. Results revealed that the majority of participants (83.4%) had a positive attitude towards people living with HIV and AIDS. This differs from previous findings where the majority indicated negative attitudes towards people living with HIV and AIDS.^22^ Positive attitudes such as these have been attributed to parental and social communication aimed at promoting HIV and AIDS awareness among the youth.^24^

### Limitations

There were a disproportionate number of female students, compared to male students in this study. It is possible that this might have affected the robustness of the chi-square test and therefore skewed the findings. In addition, the sample came from one private higher education institution in Johannesburg, South Africa. It would have been ideal for more institutions to be included in the study to get a better picture of the knowledge levels, attitudes and behaviours towards HIV and AIDS at private higher education institutions in the Johannesburg metropolitan area. Notwithstanding these limitations, these findings could serve as a springboard for national research that would be more representative of the wider student population in both private and public higher education institutions.

### Recommendations

It is recommended that future research should sample students from several private and public higher education institutions with a representative number of both male and female students. This could then inform interventions that reduce HIV infection among the youth in South Africa through improved attitudes facilitated by communication at both social and parental levels.^24^ It is further recommended that sex education in secondary schools should be introduced to close the gender gap in knowledge and prevent risky sexual behaviours.

## Conclusion

This study found that the level of HIV and AIDS knowledge in female students was not significantly different than in male students although risky sexual behaviour in female students was more frequent. In addition to the existing interventions aimed at reducing the prevalence of HIV and AIDS among the youth in South Africa, efforts towards implementing interventions at educational as well as social and family-based levels are imperative.
